# Micromolar Experiments, Nanomolar Environments

**DOI:** 10.1021/acs.est.6c13273

**Published:** 2026-10-17

**Authors:** Penghui Du, Ching-Hua Huang

**Affiliations:** † Department of Earth and Environmental Sciences, 26451 The Chinese University of Hong Kong, Shatin, Hong Kong SAR 999077, China; ‡ School of Civil and Environmental Engineering, 1372 Georgia Institute of Technology, Atlanta, Georgia 30332, United States

**Keywords:** micropollutants, trace organic contaminants, advanced oxidation processes, reactive species, oxidation kinetics, concentration dependence, transformation products, environmental fate, dissolved organic matter

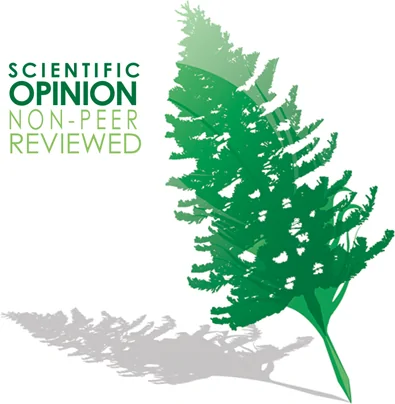

Oxidation plays an important role in the environmental fate of many micropollutants (e.g., pharmaceuticals, pesticides, and steroid hormones) by determining how rapidly and through which pathways they are transformed in waters.^1^ This chemistry spans sunlit natural waters and engineered systems such as disinfection and advanced oxidation processes (AOPs). Much of our current understanding of these processes comes from laboratory studies. For the sake of analytical convenience and kinetic resolution, laboratory oxidation experiments commonly employ micromolar target concentrations (e.g., 1–10 μM), whereas the same micropollutants generally occur at nanomolar or lower levels in real waters.^2^ Extrapolation across this concentration gap assumes that laboratory findings remain valid at real-world concentrations, an assumption rarely tested explicitly, yet one that underpins fate modeling, treatment design, and hazard assessment. Can laboratory micromolar experiments represent nanomolar oxidation in real waters? Not necessarily. The answer depends on whether the chosen initial micropollutant concentration, [MP]_0_, alters the oxidation chemistry itself, and a growing body of evidence indicates that it can.^3–7^

## When Observed Kinetics Become Concentration-Dependent

Kinetics is where the mismatch shows up first. The reaction of a micropollutant with an oxidant is commonly described by the second-order rate law −d­[MP]/d*t* = *k*[ox]­[MP], where *k* is the second-order rate constant. If [ox] remains effectively constant over the time scale of micropollutant decay, the reaction becomes pseudo-first-order, with observed rate constant *k*_obs_ = *k*[ox] and micropollutant half-life *t*_1/2_ = (ln 2)/*k*_obs_ = 0.69/(*k*[ox]).^1^ Because [MP]_0_ appears in neither expression, this routinely adopted treatment makes *k*_obs_ and *t*_1/2_ appear to be independent of the chosen initial concentration. Within an individual experiment, the constant-[ox] assumption may be reasonable. Conventional oxidants such as ozone and chlorine are often dosed in excess, whereas transient reactive species (RS) in natural photochemistry and AOPs are commonly treated at steady state, yielding a nearly constant [RS]_SS_ over the kinetic window.^1,8,9^ However, constancy within one experiment does not establish invariance across experiments conducted at different [MP]_0_ values. When varying [MP]_0_ perturbs the effective [ox], *k*_obs_ and the inferred *t*_1/2_ become concentration-dependent.^3,5^

## The Target as a Sink or Source

The target is not always a passive substrate. It can perturb [ox] as either a sink or a source (Figure 1). In the more familiar sink case, an increase in [MP]_0_ increases oxidant demand. For a conventional oxidant, once target-induced consumption becomes appreciable relative to the oxidant dose, the effective [ox] may differ across experiments. For a transient RS, its steady-state concentration, [RS]_SS_, reflects the balance between formation and total loss: [RS]_SS_ = *R*_RS_/(*k*_d_ + *k*_MP_[MP]), where *R*_RS_ is the reactive-species formation rate, *k*_MP_ is the second-order rate constant for its reaction with the target MP, and *k*_d_ is the overall pseudo-first-order decay rate constant for all other processes, including intrinsic decay and background scavenging. At a sufficiently low [MP]_0_, *k*_MP_[MP] ≪ *k*_d_, so [RS]_SS_ and the corresponding contribution to *k*_obs_, *k*_MP_[RS]_SS_, are largely insensitive to changes in [MP]_0_. As [MP]_0_ increases and *k*_MP_[MP] becomes comparable to or exceeds *k*_d_, [RS]_SS_ and the corresponding contribution to *k*_obs_ decrease.^3,9^ In sink-dominated systems, this balance gives an approximate crossover concentration, [MP]_cross_ ≈ *k*_d_/*k*_MP_, where target scavenging matches all other RS decay processes combined.^3^ Analogous estimates can guide probe and quencher dosing.^9^ Long-lived RS with low *k*_d_ and appreciable reactivity toward the target may therefore be disproportionately suppressed at high [MP]_0_ values yet become more important at low [MP]_0_ values.^3,5,6,8^ For example, Remke et al. reported that the *k*_obs_ for 3,4-dimethoxyphenol transformation photosensitized by Suwannee River fulvic acid was approximately an order of magnitude higher at an initial concentration of 0.1 μM than at 5 μM, consistent with reduced quenching of long-lived photooxidants.^5^ A *k*_obs_ measured at a micromolar [MP]_0_ can consequently underestimate the corresponding rate constant at trace concentrations.

**Figure 1:**
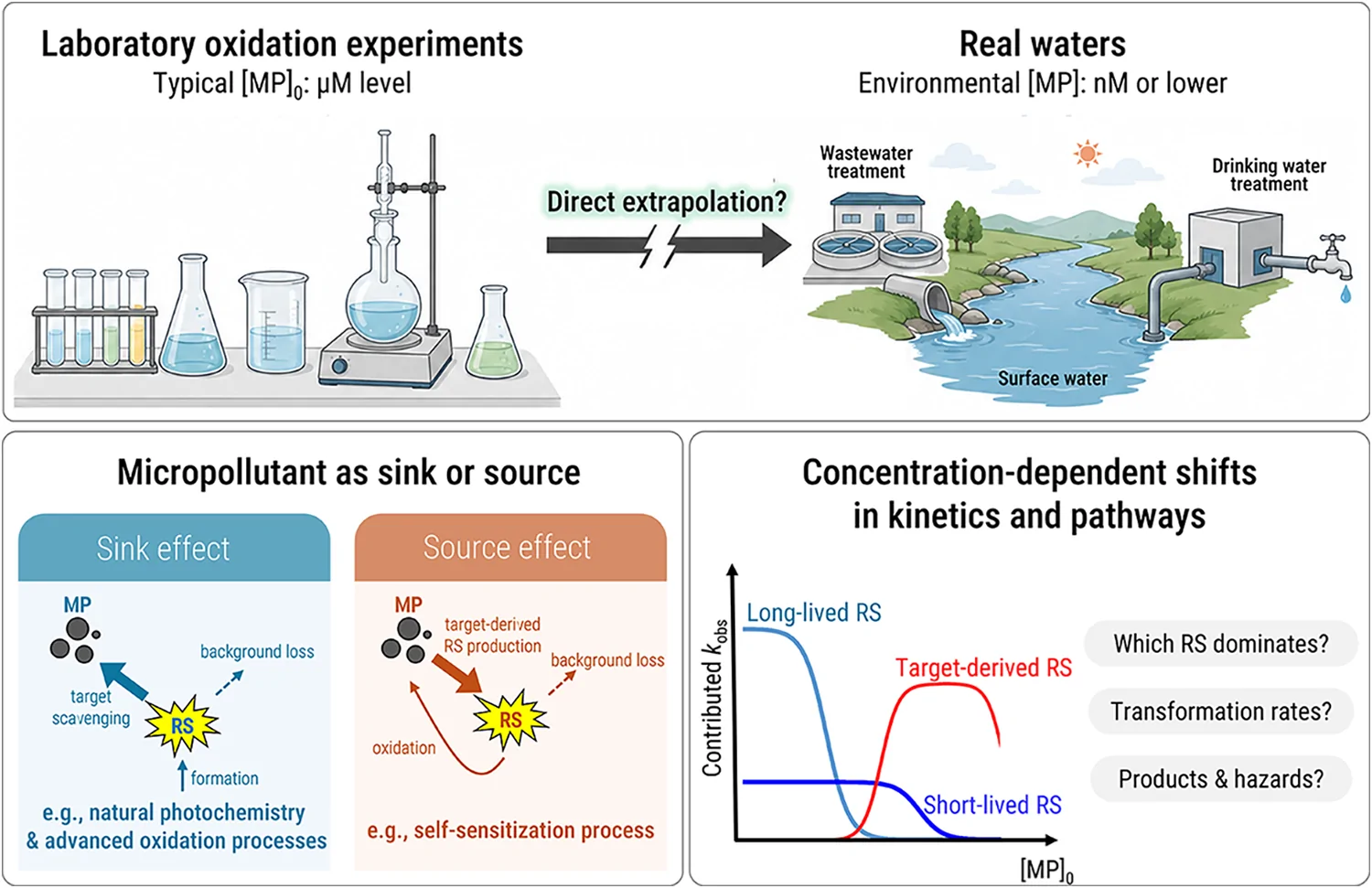
Conceptual framework for the concentration-dependent extrapolation of micropollutant oxidation from laboratory experiments to real waters. Abbreviations: MP, micropollutant; RS, reactive species; [MP]_0_, initial micropollutant concentration; *k*_obs_, observed rate constant.

A source effect, less widely recognized but consequential, produces the opposite bias. When the target micropollutant generates RS that contribute to its own oxidation, reactive-species production and hence the apparent first-order decay rate constant can increase with [MP]_0_ over a source-dominated concentration range (Figure 1).^4,7,10^ Dilution therefore weakens the source itself. 20α-Dihydroprogesterone (20α-DHP) illustrates this behavior through self-sensitized UV photodegradation, which exhibits a bell-shaped concentration response.^4^ Photoexcited 20α-DHP reduces O_2_ to superoxide, a predominant RS; dilution within the source-dominated range weakens this pathway, with the slowest initial decay observed at trace levels. In such cases, extrapolating a micromolar *k*_obs_ would instead overestimate trace-level degradation.

## Shifting Dominance, Shifting Products

Kinetics is only the beginning; reaction pathways may shift as well (Figure 1). Natural and engineered systems commonly contain multiple RS generated either directly by the process or through secondary reactions involving water constituents such as O_2_, dissolved organic matter (DOM), and carbonate species.^1,8,9^ When these species coexist, the target concentration may determine not only the overall oxidation rate but also which RS, and therefore which pathways, dominate. Because coexisting RS differ in *k*_d_ and *k*_MP_, their [RS]_SS_ and thus their respective contributions to *k*_obs_ can respond differently to changes in [MP]_0_.

For selected micropollutants in DOM-involved photooxidation, a higher [MP]_0_ can strongly suppress the steady-state concentrations of long-lived RS (e.g., DOM-derived oxidative organic radicals) through target scavenging, while those of short-lived RS (e.g., triplet-state DOM) may remain relatively unaffected.^3^ Consequently, short-lived RS may dominate oxidation at higher [MP]_0_ values, whereas long-lived RS become more important as target scavenging becomes weaker at lower [MP]_0_ values, as supported by kinetic analyses in multiple studies.^3,5,6,8^ For example, Jiang et al. treated the contribution of long-lived oxidative organic radicals to azithromycin photodegradation in dissolved black carbon solutions as negligible at 5 μM but estimated a contribution of approximately 70% at 0.1 μM.^6^ Reactive-species dominance should therefore be interpreted in the context of micropollutant concentration and reaction conditions, rather than as an intrinsic property of the process.

Reactive species also differ in how they react with micropollutants, through routes such as electron transfer, radical addition, hydrogen atom abstraction, or energy transfer.^8^ A shift in the dominant species can therefore alter the sites of attack and transformation products, with potential consequences for their biological effects.^1,3,4^ Accordingly, assuming that pathways and product profiles persist across concentration windows may compromise predictions of product formation and the associated hazard assessment.

## Practices for Concentration Extrapolation

These concentration-dependent effects call for [MP]_0_ to be treated explicitly in experimental design and interpretation. Three practices are particularly important. First, report *k*_obs_ and individual reactive-species contributions together with the [MP]_0_ at which they were determined. Second, test multiple [MP]_0_ values extending toward real-water concentrations, using sensitive detection, postquench enrichment, or constrained kinetic models where needed. Third, when concentration dependence is observed, identify its mechanistic origin and the concentration regimes over which kinetics, reactive-species dominance, or transformation pathways change. When laboratory data are used to predict environmental fate, treatment performance, or hazard, their transferability across concentration regimes should be tested rather than assumed.
